# miRNA Profile Based on ART Delay in Vertically Infected HIV-1 Youths Is Associated With Inflammatory Biomarkers and Activation and Maturation Immune Levels

**DOI:** 10.3389/fimmu.2022.878630

**Published:** 2022-04-22

**Authors:** Laura Tarancon-Diez, Irene Consuegra, Elena Vazquez-Alejo, Ricardo Ramos-Ruiz, José Tomás Ramos, María Luisa Navarro, Mª Ángeles Muñoz-Fernández

**Affiliations:** ^1^Immunology Section, Laboratorio InmunoBiología Molecular (LIBM), Hospital General Universitario Gregorio Marañón, Madrid, Spain; ^2^Instituto de Investigación Sanitaria Gregorio Marañón (IiSGM), Area of Immune System Pathology, Madrid, Spain; ^3^Genomic Facility, Parque Científico de Madrid, Madrid, Spain; ^4^Department of Paediatrics, Clínico San Carlos University Hospital, Madrid, Spain; ^5^Pediatric Infectious Disease Unit, Hospital Gregorio Marañón, Instituto de Investigación Sanitaria Gregorio Marañón (IiSGM), Universidad Complutense de Madrid and CIBERINFEC, Madrid, Spain; ^6^Universidad Complutense de Madrid, Madrid, Spain

**Keywords:** miRNA profile, vertically acquired-HIV-1 infection, ART, youths, inflammatory profile

## Abstract

Early antiretroviral treatment (ART) in vertically acquired HIV-1-infection is associated with a rapid viral suppression, small HIV-1 reservoir, reduced morbimortality and preserved immune functions. We investigated the miRNA profile from vertically acquired HIV-1-infected young adults based on ART initiation delay and its association with the immune system activation. Using a microRNA panel and multiparametric flow cytometry, miRNome profile obtained from peripheral blood mononuclear cells and its association with adaptive and innate immune components were studied on vertically HIV-1-infected young adults who started ART early (EARLY, 0-53 weeks after birth) and later (LATE, 120-300 weeks). miR-1248 and miR-155-5p, were significantly upregulated in EARLY group compared with LATE group, while miR-501-3p, miR-548d-5p, miR-18a-3p and miR-296-5p were significantly downregulated in EARLY treated group of patients. Strong correlations were obtained between miRNAs levels and soluble biochemical biomarkers and immunological parameters including CD4 T-cell count and maturation by CD69 expression on CD4 T-cells and activation by HLA-DR on CD16^high^ NK cell subsets for miR-1248 and miR-155-5p. In this preliminary study, a distinct miRNA signature discriminates early treated HIV-1-infected young adults. The role of those miRNAs target genes in the modulation of HIV-1 replication and latency may reveal new host signaling pathways that could be manipulated in antiviral strategies. Correlations between miRNAs levels and inflammatory and immunological markers highlight those miRNAs as potential biomarkers for immune inflammation and activation in HIV-1-infected young adults who initiated a late ART.

## Introduction

Antiretroviral treatment (ART) implementation has greatly reduced mother to child HIV-1 transmission. Despite this, at the end of 2019, 1.7 million people living with HIV-1 were children aged 0 to 14 years old (1). WHO guidelines recommend that ART should be initiated within 1 year of age in all vertically acquired HIV-1 infected children as it is well known that the earlier the ART is initiated after infection, the greater the reduction in the reservoir and disease progression, not only in HIV-1 infected children but also in adults (2–4). Several observations suggested that a late timing of treatment initiation in HIV-1 infected children is associated with the induction of ineffective HIV-specific immune responses (5, 6), altered immune phenotype (7) and a distinct transcriptional signature and protein profile, showing upregulation of immune activation pathways (5, 8), indicated a proinflammatory state that may potentially trigger premature non-AIDS events including atherosclerotic and metabolic diseases. It is still unclear whether the effects of the infection on an immune system under development are reversible later in life, as first children infected since birth are now reaching adulthood. Immunological data on this population remain scarce, but our group along with others has described a premature immune aging profile associated with HIV-1 infection (7, 9).

MicroRNAs (miRNAs) are small noncoding RNAs implicated in the development and progression of multiple diseases and have recently been proposed as therapeutic targets in cancer, cardiovascular and metabolic diseases and infections due to their crucial roles in processes of cell maturation, proliferation, migration, differentiation and function in the immune system (10, 11). Focusing on HIV-1 infection, there is a strong effort centered on determining how HIV-1 can differentially modulate several miRNAs that potentially regulate host cellular pathways such as cell cycle, apoptosis, T-cell signalling and cytokine response (12, 13). Because of that, cellular miRNAs could possibly be involved in establishing HIV-1 latency. Recent strategies for clearing viral reservoir have resulted from studies on the mechanism of how miRNAs influence viral protein expression (14). Up to the moment, most findings related to miRNA in HIV-1-infection have been obtained from patients infected during adulthood focussing on the disease progression and there is no information on the miRNA profile related to an early ART initiation in vertically infected HIV-1-patients that reached adult status. Thus, the objective of the present preliminary study was to investigate the miRNA expression profile from vertically acquired HIV-1-infected young adults based on ART initiation delay as well as its association with inflammatory biomarkers and immune system activation and maturation state.

## Materials and Methods

### Study Participants

This cross-sectional study is based on young adults with vertically transmitted HIV-1 infection from the Paediatric Spanish AIDS Research Network Cohort (coRISpe). All participants were Caucasian. Eighteen patients, all of them on suppressive ART treatment, were included based on sample availability and the following inclusion criteria: 1) patients had evidence of virological suppression within the first year after ART initiation; 2) had subsequent maintenance of viral control (≤20 copies/mL) for at least five years before sampling; 3) were not coinfected by Hepatitis C Virus (HCV). Cryopreserved peripheral blood mononuclear cells (PBMCs) and associated clinical data were provided by the Spanish HIV Hospital General Universitario Gregorio Marañón BioBank (15) and by coRISpe (16) respectively. Patients were classified in two groups according to ART timing initiation in those who initiated ART early (EARLY, 0-53 weeks, n=7) and those who initiated ART late (LATE, 120-300 weeks, n=11). As reference group, samples of fresh whole blood from volunteers non-HIV-1-infected Healthy Donors (HD, n=6) paired by sex and age were collected in EDTA tubes and PBMCs were immediately isolated by Ficoll-Paque density gradient centrifugation and stored at -20°C until RNA extraction.

The study was approved by the ethic committee of Hospital General Universitario Gregorio Marañón (HGUGM) in Madrid (protocol miRNA-Ped-HIV_16, approved on March 21, 2017, acta06/2017). Written informed consent was obtained from all participants before inclusion in coRISpe and from all volunteers before inclusion in the study. Data was collected from October 2020 to March 2021.

### Immunophenotyping of T lymphocytes and NK Cells

Immunophenotyping of T lymphocytes and Natural Killer (NK) cells using multiparametric flow cytometry was performed following this hierarchy order according to sample availability. In future analysis, the number of patients will vary due to this limitation. For T lymphocyte immunophenotyping, two flow cytometer panels were designed. For the first panel PBMCs were thawed, washed and stained with LIVE/DEAD fixable Aqua Blue Dead Cell Stain (Life Technologies, CA, USA) for viability, and the surface antibodies CD56-CD19-CD14-BV510 (CD56 clone NCAM16.2; CD19 clone SJ25C1 and CD14 clone MOP9), CD3-PerCP-Cy5.5 (clone SK7) (BD Biosciences, San Diego, CA) and CD8-PB (clone RPA-T8; Biolegend, San Diego, CA) for lineage, CD45RA-ECD (clone 2H4), IL-7 receptor, CD127-PeCy7 (clone R34.34), HLA-DR-APC (clone GRB-1) (Beckman Coulter), CD38-FITC (clone HB7), CD69-APC-R700 (clone FN50) (BD Biosciences) for maturation, survival and activation.

The second T lymphocyte panel, in addition to LIVE/DEAD fixable Aqua Blue Dead Cell Stain for viability, CD56-CD19-CD14-BV510, (CD56 clone NCAM16.2; CD19 clone SJ25C1 and CD14 clone MOP9), CD3-APC-Cy7 (clone SK7) for lineage and CD45RA-ECD (clone 2H4; Beckman Coulter) and CD27-PerCP-Cy5.5 (clone M-T271, BD, Biosciences) for maturation, it also included CD4-APC-R700 (clone RPA-T4, BD Biosciences) for lineage, CD57-FITC (clone NC1; Beckman Coulter) for senescence and TIM-3-PE (clone 7D3), PD-1 (clone EH12.1) (BD Biosciences), LAG-3-Pe-Cy7 (clone 7H2C65) and TIGIT-AF647 (clone A15153G) (Biolegend), for exhaustion. Lymphocytes were defined as viable cells having low forward/side scatter and expressing CD3, and/or no CD8/CD4, but not CD19, CD14 and CD56. Isotypes controls were included for CD57, TIM-3, LAG-3, TIGIT and PD-1.

The NK immunophenotyping included: LIVE/DEAD fixable Aqua Blue Dead Cell Stain for viability, CD3-CD19-CD14-BV510 (CD3 clone SK7; CD19 clone SJ25C1 and CD14 clone MOP9), CD56-APC-Cy7 (clone NCAM16.2), CD16-PerCP-Cy5.5 (clone 3G8) (BD Biosciences) for NK subsets identification and CD57-FITC (clone NC1) for maturation, HLA-DR-APC (clone GRB-1) (Beckman Coulter) for activation, the constitutively expressed receptor, TIM-3-PE (clone 7D3) involved in NK cell cytokine secretion, the C-type lectin-like activating receptor NKG2D-PECF594 (clone 1D11), CD69-APC-R700 (clone FN50) for activation, the inhibiting receptor NKG2A-BV421 (clone 131411) (BD Biosciences) and the natural cytotoxicity family activating receptor NKp30-PE-Cy7 (clone P30-15; Biolegend). To identify NK cells, viable cells, negative for CD3, CD14 and CD19 were classified also in three subsets based on the expression of CD56 (CD56^neg^, CD56^high^ and CD56^dim^) and CD16 that define the NK cytotoxic subset (CD16^high^) Isotype controls were included for TIM-3, NKG2D, NKG2A, NKp30 and CD69.

Acquisition was carried out in a Galios flow cytometer (Beckman Coulter). Before acquisition, cells were fixed with 4% paraformaldehyde. At least 1 million events were acquired for each condition. FlowJo software (v10.6.1) was used for data analysis.

### RNA Extraction and miRNA Profiling

Total RNA isolation was performed with mirVana miRNA isolation kit (Ambion, Huntingdon, UK), following the manufacturer’s instructions and RNA concentrations were estimated with Nanodrop ND-1000 (Thermo Scientific, Waltham, MA, USA) followed by RNA analysis of quality and integrity based on RIN values, determined using Agilent 2100 Bioanalyzer (Agilent Technologies, Santa Clara, CA, USA). TaqMan™ Advanced miRNA cDNA Synthesis Kit (Thermo Fisher) was used for reverse-transcription of isolated RNA. MiRNA gene expression profile was then assessed by using the 754 panel TaqMan™ OpenArray™ Human Advanced MicroRNA Panel and run in the Open Array block of a QuantStudio™ 12K Flex equipment (ThermoFisher).

The RQ module (version4.3) of the apps.thermofisher cloud (apps.thermofisher.com) was used to generate Crt values (herein referred as Cq) and to calculate deltaCq (∆Cq) data using Global Normalization. Cq values ≥ 32 were considered negative and microRNAs which did not show expression above this level in at least 12 out of the 24 samples were discarded. Therefore, 142 miRNAs were detected as positive in this study. MiRNA expression profile analysis from six healthy donors non-HIV-infected subjects, paired by sex and age with HIV-1 participants, was also included. Reactions and data analysis were carried out at Genomics Core Facility, Parque Científico de Madrid, Spain.

### Statistical Analysis

Shapiro-Wilk normality test was used for variables normality and they were non-normally distributed and then considered as non-parametric. Differences between categorical and continuous values were determined by the Chi-square test and Mann-Whitney U test, respectively. Correlations between variables were assessed using the Spearman rank test. *p* values <0.05 were considered statistically significant.

The statistical software used included SPSS 23.0 package (IBM, Madrid, Spain). Graphs were generated with Prism, version 9.0 (GraphPad Software, Inc.).

## Results

### General and Clinical Characteristics of the Studied Subjects

Clinical and demographic characteristics of patients are shown in **Table 1**. Comparing groups, statistical difference was only observed in age at sampling, being LATE group older than EARLY group (24 [22-25] and 21 [20-23] years respectively, *p*=0.035). No differences were found in the clinical and immunological parameters including CD4+ and CD8+ percentage and cell counts, CD4+/CD8+ ratio, nadir CD4+ cell count, time since HIV-1 diagnosis, time under ART and under virological control.

**Table 1:** Study cohort characteristics.

	Treatment Initiation		*P*
	EARLY (0-53 weeks) n=7	LATE (120-300 weeks) n=11	
**Subject characteristics**			
Age at ART initiation (wk)	35 [31-47]	237 [200-264]	**<0.001**
Sex (male) n. (%)	3 (43)	5 (54.5)	0.914
Nadir CD4+ cells/mm^3^	282 [50-432]	158 [88-327]	0.342
**Parameters at sampling**			
Age (years)	21 [20-23]	24 [22-25]	**0.035**
Time since ART initiation (years)	20 [20-22]	19 [17-23]	0.315
Time under virologic control (years)	10 [5-11]	9 [7-13]	0.682
Time since HIV diagnosis (years)	20 [20-22]	22 [19-24]	0.383
n CD4+ cells/mm^3^	903 [781-1166]	780 [598-924]	0.113
n CD8+ cells/mm^3 a^	602 [512-1028]	784 [751-1028]	0.491
Ratio nCD4+/nCD8+ ^a^	1.5 [0.85-2.24]	0.98 [0.65-1.08]	0.153
%CD4	42 [33-49]	36 [31-39]	0.188
%CD8 ^a^	28 [25-39.8]	38 [33.5-46]	0.099
Ratio %CD4/%CD8 ^a^	1.5 [0.85-2]	1 [0.65-1.08]	0.223

Values are show as median [IQR] for continuous variables or num (%) for categorical variables. Mann-Whitney test and Chi-square test were used for comparisons between continuous and categorical variables respectively. Significant values are shown in bold. ART, antiretroviral therapy; wk, weeks. ^a^Data available for 16 patients.

Biochemical and inflammatory biomarkers including plasma levels of glucose, GPT (ALT), GOT (AST), bilirubin, total cholesterol, triglycerides, HDL, LDL, LDL/HDL ratio and creatinine at sampling are showed in **Table 2**. EARLY group showed increased levels of triglycerides (*p*=0.044) and a trend to low frequency of HDL (*p*=0.097).

**Table 2:** Biochemical and inflammatory biomarkers.

	Treatment Initiation		*p*
	EARLY (0-53 weeks) n=7	LATE (120-300 weeks) n=11	
**Glucose (mg/dL)**	84 [77-90]	83 [73-94]	0.786
**GPT (ALT) U/L**	22 [17-56]	20 [13-39]	0.496
**GOT (AST) U/L**	23 [18-42]	19 [19-28]	0.492
**Bilirubin (mg/dL)**	0.57 [0.39-0.9]	0.34 [0.25-0.56]	0.103
**Total cholesterol (mg/dL)**	172 [153-202]	158 [145-164]	0.211
**Triglycerides (mg/dL)**	159 [114-226]	88 [52-126]	**0.044**
**LDL (mg/dL)^a^**	93 [81.2-111]	89 [76.7-113]	0.958
**HDL (mg/dL)^b^**	44.2 [43-47]	52.5 [47-61.25]	0.097
**LDL/HDL^a^**	2.02 [1.56-2.58]	1.64 [1.22-2.47]	0.315
**Creatinine (mg/dL)**	0.72 [0.54-0.78]	0.74 [0.71-0.86]	0.496

Variables are taken at sampling. Values are shown as median [IQR] and statistical differences are using the Mann-Whitney test. Significant values are shown in bold. ALT, Alanine Aminotransferase; AST, Aspartate Aminotransferase; GOT; Glutamic Oxaloacetic Transaminase; GPT, Glutamate Pyruvate Transaminase; HDL, high-density lipoprotein cholesterol; LDL, low-density lipoprotein cholesterol. ^a,b^Data available for 16 and 17 patients respectively.

### Altered miRNA Expression Profile in Late Treated Vertically HIV-1-Infected Young Adults

The miRNA profiling analysis in PBMCs detected and quantified 142 miRNAs. Comparisons between HD (median age of 23 years, 50% male) and EARLY and LATE treated HIV-1-infected young adults showed 34 and 28 differentially expressed miRNAs respectively (see **Supplementary Table 1**).

**Figure 1:**
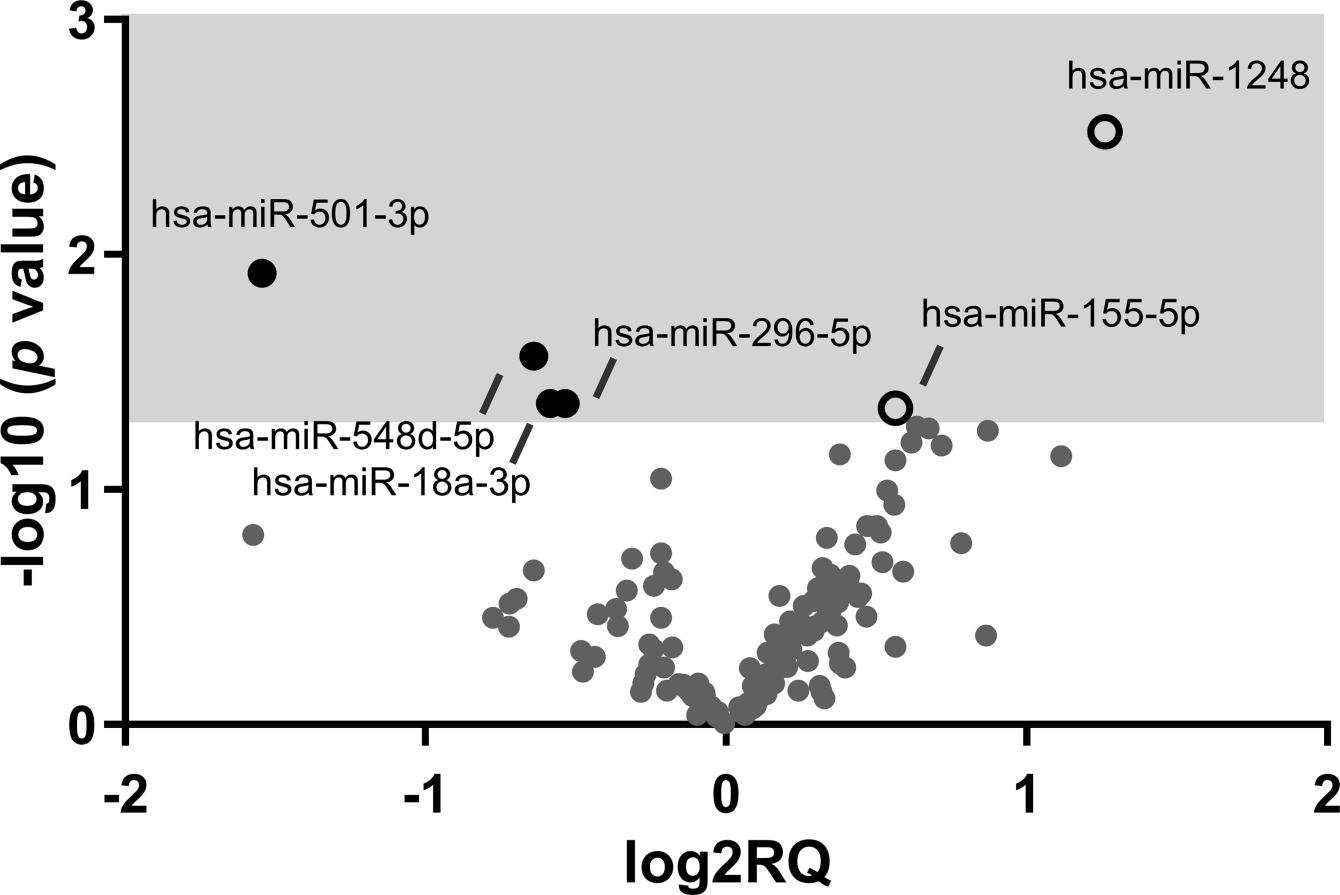
Volcano plot showing the results of the miRNA profile analysis comparing EARLY and LATE-treated groups of HIV-infected patients. Graph of log2 Relative Quantity ratio (log2RQ) against log10 p value significance (-log10 *p* value) from 142 quantified miRNAs. Open white spots represent upregulated genes and black spots represent downregulated genes on EARLY treated (n=7) compared to LATE (n=11) treated HIV-1-young adults.

Comparing both groups of HIV-infected young adults, six miRNAs were expressed differently: two were significantly upregulated in EARLY group compared with LATE group (miR-1248 and miR-155-5p) and four (miR-501-3p, miR-548d-5p, miR-18a-3p and miR-296-5p) were significantly downregulated in EARLY treated group of patients (**Figure 1**). Noteworthy, one of that subset of regulated miRNAs (miR-501-3p) was also found modulated in HD group compared with both groups of HIV-infected young adults. Focusing on EARLY versus LATE comparison, for target genes identification, the public database miRTarBase was accessed because it gathers functional studies of miRNA–target interactions, which are validated experimentally (http://mirtarbase.cuhk.edu.cn/) and genes with strong evidence of interactions when linked to targets related to HIV-1 infection based on information available in scientific literature. Regulated genes involved in HIV-1 infection by miR-18a-3p, miR-296-5p and miR-155-5p are shown in **Table 3** while no evidence was found for miR-1248, miR-501-3p or miR-548d-5p.

**Table 3:** Differentially expressed miRNAs between LATE and EARLY treated HIV-1-young adults and list of targeted genes involved in HIV immunopathogenesis.

miRNA	P value	LATE/EARLY– ratio	Genes	Proteins	Cites*
miR-1248	0.003	2.395	*NE*		
miR-501-3p	0.012	0.343	*NE*		
miR-548d-5p	0.027	0.642	*NE*		
miR-18a-3p	0.043	0.667	CBX7	Chromobox protein homolog 7	(17)
miR-296-5p	0.043	0.680	PIN1	Peptidylprolyl Cis/Trans isomerase NIMA-Interacting-1	(18)
			PLK1	Polo-like kinase 1	(19)
miR-155-5p	0.045	1.477	MECP2	Methyl-CpG Binding Protein 2	(17)
			SOCS1	Suppressor of cytokine signaling 1	(20)
			HIF1A	Hypoxia inducible factor 1 alpha (HIF-1-alfa)	(21)
			MYO10	Myosin-X	(22)
			POLE3	DNA polymerase epsilon subunit 3	(21)
			ARID2	AT-rich interactive domain-containing protein 2	(23)
			MATR3	Matrin-3	(24)
			RHOA	Ras homolog family member A (RhoA)	(25)
			FOXO3	Transcription factor forkhead box O-3 (FoxO3)	(26)
			PSIP1	Cellular chromatin-associated protein LEDGF/p75	(27)

The reference group in the comparison is LATE group. *References to experimental evidence of gene expression regulation by miRNAs and relation of those proteins in HIV pathogenesis are provided. NE, No Evidence.

**Figure 2:**
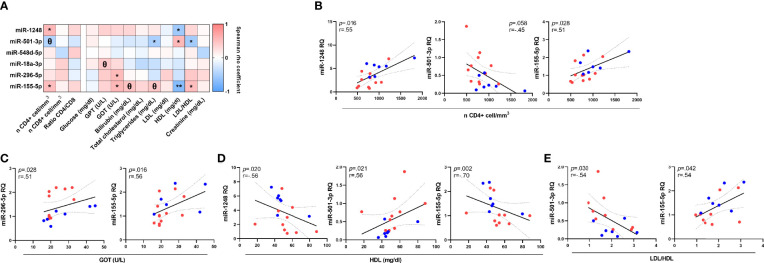
Associations between miRNA relative levels and immunological and biochemical parameters on HIV-1-infected young adults. Correlations of miRNAs relative quantity (RQ) with all soluble immunological and biochemical parameters **(A)**. Significant and close to significant associations with T-CD4+ cell count **(B)**, Glutamic Oxaloacetic Transaminase (Aspartate Aminotransferase) (GOT; AST) **(C)**, high-density lipoprotein cholesterol (HDL) (data available for 16 patients) **(D)** and LDL/HDL ratio (LDL: low-density lipoprotein cholesterol) (data available for 16 patients) **(E)**. Patients from EARLY treated group (n=7) are highlighted with blue dots and red dots represent LATE treated HIV-1-young adults (n=11). The Spearman rho correlation coefficient test was used. **p <0.01, *p <0.05, ^Θ^0.05<*p*< 0.1.

To determine possible associations between miRNAs profile and clinical and immunological parameters on HIV-1 infected patients, the levels of six most significant miRNAs were correlated with immunological and biochemical parameters (**Figure 2A**). The relative levels of miR-1248, miR-501-3p and miR-155-5p were associated with CD4+ cell counts (*p*=0.016; ρ=0.55; *p*=0.058; ρ=-0.58 and *p*=0.028; ρ=0.51 respectively). Besides, miR-296-5p and miR-155-5p correlated directly with soluble GOT transaminase (*p*=0.028; ρ=0.51 and *p*=0.016; ρ=0.56 respectively) and concerning soluble lipoproteins, strong associations were found between HDL soluble levels with miR-1248, miR-501-3p and miR-155-5p relative levels (*p*=0.020; ρ=-0.56; *p*=0.021; ρ=0.56 and *p*=0.002; ρ=-0.70 respectively) and ratio LDL/HDL with miR-501-3p and miR-155-5p relative levels (*p*=0.030; ρ=-0.54 and *p*=0.042; ρ=0.54 respectively) (**Figures 2B–E**).

### Activation and Exhaustion Levels on T Cells and NK Cells and the Relationship With miRNAs Expression in Vertically Acquired HIV-1 Young Adults

**Figure 3:**
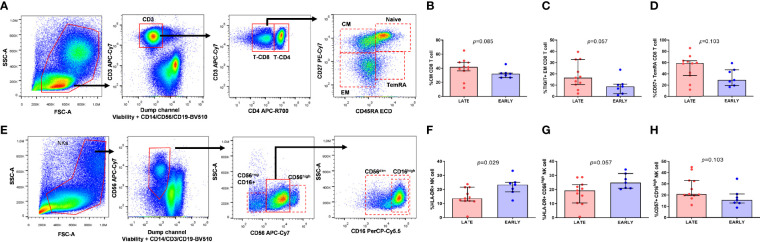
Memory CD8-T cell distribution and activation and exhaustion expression markers on CD8-T cell subsets and NK cell subsets from HIV-infected young adults. Gate strategy for memory subset distribution on T cell **(A)**, lymphocytes were characterized by the patterns of forward and side scatter, followed by selection of CD3+. Central Memory (CM) (CD45RA-CD27+) CD8 T cell subset distribution **(B)**, TIGIT expression on Effector Memory (EM) (CD45RA-CD27-) CD8 T cell subset **(C)** and CD57 expression on Terminally Differentiated (TemRA) (CD45RA+CD27-) CD8 T cell subset **(D)**. Gate strategy for NK cell subsets **(E)** viable cells, negative for CD3, CD14 and CD19 were classified in three subsets according to the expression of CD56 and CD16. HLA-DR expression on total NK cells **(F)** and CD56^high^ **(G)** NK cell subset and CD57 expression on CD16^high^ NK cell subset **(H)** from EARLY treated group (n=7) and LATE treated HIV-1-young adults (n=11). Mann-Whitney U test was used.

CD4 and CD8 T-cell maturation subsets were defined as naïve (CD45RA+CD27+), Central Memory (CM; CD45RA-CD27+), Effector Memory (EM; CD45RA-CD27-) and Terminally Differentiated (TemRA; CD45RA+CD27-) (gate strategy in **Figure 3A**) and NK cells were classified also in subsets based on the expression of CD56 (CD56^neg^, CD56^high^ and CD56^dim^) and CD16 that define the NK cytotoxic subset (CD16^high^) (gate strategy in **Figure 3E**).

Firstly, we wanted to determine if EARLY and LATE treated HIV-1 patients showed differences in subset distribution and activation, survival and exhaustion levels on T and NK cells. Only borderline statistical significance was observed, probably due to the scarce sample size, towards a higher frequency of CM CD8 T-cells and TIGIT and CD57 expression on EM and TemRA CD8 T-cells from LATE group, respectively (**Figures 3B–D**). Regarding NK cells, gate strategy is defined in **Figure 3E**, LATE group had significant lower activation levels defined by HLA-DR on total NK cells (*p*=0.029) and a borderline statistical significance on CD56^high^ NK subset and higher CD57 levels on CD16^high^ NK subset compared to EARLY group (**Figures 3F–H**).

**Figure 4:**
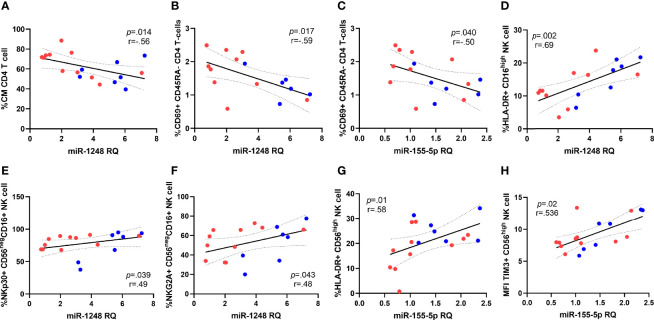
Correlations between miRNAs and subset distribution and activation markers expression on CD4 T cells and NK cells. Correlations of miRNA-1248 relative quantity (RQ) with CD4 Central Memory subset distribution (CM, defined as CD45RA-CD27+) **(A)** and CD69+ expression on CD45RA- CD4 T cells **(B)**, data available for 16 patients. MiRNA-155-5p correlations with CD69+ expression on CD45RA- CD4 T cells **(C)**, data available for 16 patients. Correlations of miRNA-1248 levels and expression of HLA-DR on CD16^high^ NK subset **(D)** and NKp30 and NKG2A on CD56^neg^CD16+ NK cell subset **(E, F)**. Correlations of with miRNA-155-5p levels with HLA-DR expression on CD56^high^ NK cells **(G)** and median intensity fluorescence (MFI) of TIM-3 expression on CD56^high^ NK cell subset **(H)**. Patients from EARLY treated group (n=7) are highlighted with blue dots and red dots represent LATE treated HIV-1-young adults (n=11). The Spearman rho correlation coefficient test was used.

We next analyzed the relationship between T and NK cell activation, survival and exhaustion levels and miRNAs expression, including those six that discriminate both groups of HIV-patients. Regarding T cells, CM CD4 T-cell subset distribution negatively correlated with miRNA-1248 relative levels (**Figure 4A**). Besides, expression levels of activation marker CD69 on naïve CD4 T-cells (defined as CD45RA+) was inversely correlated with miRNA-1248 relative levels, (ρ=-0.52; *p*=0.020) and similarly, CD69 expression levels on memory CD4 T-cells (defined as CD45RA-) also negatively correlated with miRNA-1248 and miRNA-155-5p relative levels (**Figures 4B, C**). No correlations were found for exhaustion markers on CD4 T-cells, neither for CD8 T-cell markers.

Focusing on NK cell associations, miRNA-1248 levels strongly and directly correlated with the expression of the activation marker HLA-DR on total NK cells subsets (ρ=0.569; *p*=0.014) and that association remains significant on the cytolytic NK subset CD16^high^ expressing HLA-DR and miRNA-1248 (**Figure 4D**). Besides, miRNA-1248 levels also correlated with the expression of NKp30 and NKG2A on CD56^neg^CD16+ NK cells subset (**Figures 4E, F**). In the case of miRNA-155-5p, upregulated on EARLY treated group, its levels strongly and directly correlated with HLA-DR expression and MFI of TIM-3 expression on CD56^high^ NK cell subset (**Figures 4G, H**). Most of those associations were lost after stratification by group probably a cause of the small number of studied cases (data not shown).

## Discussion

In this work, for the first time we describe a specific miRNA signature on peripheral blood mononuclear cells of vertically HIV-1-young adults who started an ART during the first year after birth compared to late treated HIV-1-young adults, with the purpose of performing a distinctive screening of miRNAs that potentially control the expression of multiple related genes in the context of HIV-1 control and future delayed progression due to their relevant role in HIV-1 cycle and in the establishment of reservoirs. Besides, the miRNome assay was completed with an in-depth phenotype characterization on innate and adaptive immune system components. The associations found between miRNAs and biochemical inflammatory biomarkers and activation and maturation markers expression highlight the potential role of those miRNAs as biomarkers for immune activation and can be considered good targets for therapies aimed at modulating immune response in HIV-1-young adults.

Our study confirms previous studies in which HIV-1 infection leads to altered miRNome compared to non-HIV-1 infected population. However, all evidences were described on HIV-1 patients infected at adulthood by sexual route among other ways of transmission and tried to find patters related to immune and viral progression (28). Up to the moment, none information had been carried out from vertically acquired HIV-1 infected group that still represents more than 150,000 new infections worldwide every year (1).

Focusing on most relevant identified miRNAs comparing EARLY and LATE treated HIV-1-young adults, miRNA-155-5p has been widely studied due to its role in the functionality of the immune system regulating the immune response and lymphocyte differentiation (29, 30). Besides, miRNA-155 has been highlighted as potential biomarker of long-term nonprogressors (LTNPs) who showed decreased levels compared to viremic naïve HIV-subjects suggesting an important role also in the control and pathogenesis of HIV-1 infection (31). Accordingly to that, but contrary to what we expected due to all well-known benefits associated to an EARLY antiretroviral therapy introduction described up to the moment (2, 4, 32, 33), our group of LATE treated patients would look like LTNPs. In LTNP patients, an increased adaptive immune activation has been described suggesting that spontaneous viral control may imply an immunological cost in those patients who maintain virologic control in the absence of treatment. Similarly, the activated immune phenotype we described herein on LATE treated vertically HIV-1 patients with higher viral reservoir, could reflect the exacerbated immune pressure needed against viral replication on this group. Interestingly, increased levels of miRNA-155 have been correlated with increased immune activation in HIV-scenario (34) accordingly to our results.

Regarding miRNA-18a, despite scarce evidences related to miR-18a-3p, low levels of miRNA family members like miRNA-18a and miR-18b-5p in LTNP compared to typical progressors indicated the potential role of that miRNA in viral replication and disease progression (35) by controlling genes of the inflammatory response as HIF-1α (36). The low levels of miRNA-18a-5p expression observed in EARLY treated vertically acquired HIV-patients studied in this work could explain the low viral reservoir and immune activation that has widely characterized this group of patients (37).

In addition to everything described so far related to miRNA-155-5p, that miRNA and miRNA-1248, which is associated with good survival prognoses in cancer (38), both upregulated on EARLY treated HIV-1 patients, strongly correlated with the activation marker HLA-DR, the natural cytotoxicity family activation receptor NKp30 and the inhibiting receptor NKG2A expressed on NK cells and more specifically on the main cytotoxic NK subset, CD16^high^ and the activated and mature CD56^neg^CD16+ NK cells, described as a subset with impaired effector functions in HIV-1 infection (39). Despite the limitation that miRNA profile was determined in the pool of PBMCs without isolated different cell subsets, our results are in agreement with recent insights that have highlighted the role of miRNAs in maintaining killing effect of NK cells in cancer scenario *via* activation markers modulation (40–42), in promoting antiviral immunity in the scenario of viral infection (43) and altogether suggests the possible role of both, miRNA-155-5p and miRNA-1248 in regulating inflammatory cytokine production in NK cells, potentially balancing disease progression during chronic HIV infection. Higher TIM-3 expression in NK cells has been considered a marker of better prognosis during HIV infection since its down regulation is indicative of IFN-γ deficient response (44) and interestingly, strong and direct correlations were also found between miRNA-155-5p levels, miRNA upregulated on EARLY treated HIV-1 young adults, and MFI expression of TIM-3 in CD56^high^ NK cells.

MiRNAs have also arisen as critical regulators of lipidic metabolism controlling genes involved in HDL and LDL secretion, the most important risk factors of atherosclerosis, cholesterol biosynthesis and hepatic LDL receptor expression (45, 46). In our study EARLY treated HIV patients had decreased HDL levels traditionally associated with risk for developing cardiovascular diseases (CVD) and, while no differences were found in ratio LDL/HDL comparing groups, both biochemical parameters correlated with miRNA-501-3p and miRNA-155-5p relative levels. In the case of miRNA-501-3p, downregulated on EARLY treated HIV-1 young adults, that miRNA levels correlated directly with soluble HDL and inversely with ratio LDL/HDL and the opposite correlations were observed for miRNA-155-5p, upregulated on EARLY treated group.

Multiple miRNAs are being also evaluated as novel plasma biomarkers that out-perform or add value to the conventional liver injury biomarkers ALT, AST among others (47). Interestingly, miRNA-296-5p and miRNA-501-3p levels, both downregulated on EARLY group compared with LATE group, also correlated directly with AST levels. Recent evidences indicate that miRNA-296-5p regulates liver PIN1 and PLK1 genes that encode peptidylprolyl Cis/Trans isomerase NIMA-Interacting-1 and polo-like kinase 1 respectively, both involved in HIV-1 replication cycle stages (18, 19), suggesting that miRNA-296-5p may have a regulatory activity on virus replication in HIV pathogenesis and contributing to immune activation. These indicators could be potential biomarkers for disease progression and, in agreement with previous observations (48), good targets for therapies aimed at modulating the immune response in HIV infection. All explain herein, reinforce the idea that those distinct miRNAs could be considered regulators of cholesterol metabolism, hepatic lipoprotein synthesis, potential liver damage biomarkers and therapeutic targets for the treatment of CVD. However, genetic ablation of other miRNAs in the context of CVD development have led also to contradictory effects depending on animal model raising concerns about the use of therapies based on miRNAs (49).

There is unclear information on the risk of developing CVDs among other pathologies as our study is based on the time of treatment introduction and the cross-sectional design of our study as main limitation. In addition, the scarce number of participants and the low statistical power do not allow us to find differences in the immunological comparisons and results obtained here should be validated in a larger cohort of patients. Moreover, the in-depth pairing between groups in terms of CD4, CD8-T cell counts, time since ART initiation and since virological control can also explain the lack of immune phenotype differences on T- and NK cells. The panel used for miRNAs profile had limited number of them. However, samples of vertically infected patients reaching adulthood are scarce and mentioned limitations are also counterbalanced by the exhaustive follow-up of the participants during a median of 21 years since HIV diagnosis and during that period none of them has developed any inflammatory disease or non-AIDS-event. Further longitudinal observation would be necessary to determined clinical consequences that could be explained by the EARLY start of treatment.

In conclusion, our finding demonstrates that there is a miRNA signature that discriminates LATE treated from EARLY treated vertically acquired HIV-1-infected young adults. The role of those distinct miRNAs target genes in the modulation of HIV-1 replication and latency reveals new host targets that may be modulated in future antiviral strategies. Moreover, we found strong correlations between the most significant miRNAs and soluble inflammatory biomarkers and NK and T-cell activation and maturation suggesting that those miRNAs may be potential biomarkers for immune activation and progression disease in vertically acquired HIV-1-infected young adults who initiated a late ART and in general HIV-1-infected patients.

## Data Availability Statement

The raw data supporting the conclusions of this article will be made available by the authors, without undue reservation.

## Ethics Statement

The studies involving human participants were reviewed and approved by Comité de Ética de Investigación con Medicamentos. Written informed consent to participate in this study was provided by the participants’ legal guardian/next of kin.

## Author Contributions

MAM-F designed the research study. LT-D, IC, EV-A designed and performed the research studies. RR contributed essential tools. LT-D and EV-A analyzed the data. LT-D wrote the paper. JTR, MLN, and MAM-F critically discussed the paper. All authors have read and approved the final manuscript.

## Funding

LT-D is supported by the Instituto de Salud Carlos III (ISCIII) under grant agreement “CD20/00025” through the Sara Borrell Program and by GeSIDA through the “Premio para Jóvenes Investigadores 2021”. EV-A was supported by Comunidad de Madrid, Programa de Empleo Joven “PEJ-2017 AI_BMD-7446”. This work has been (partially) supported by the RD16/0025/0019 projects as part of Acción Estratégica en Salud, Plan Nacional de Investigación Científica, Desarrollo e Innovación Tecnológica (2020–2022) and co-financed by Instituto de Salud Carlos III (Subdirección General de Evaluación) and Fondo Europeo de Desarrollo Regional (FEDER), RETIC PT17/0015/0042, Fondo de Investigación Sanitaria (FIS) 2020-2022 (grant number PI19/01638) and EPIICAL Project. Moreover, this work has been supported partially by a EUROPARTNER: Strengthening and spreading international partnership activities of the Faculty of Biology and Environmental Protection for interdisciplinary research and innovation of the University of Lodz Programme: NAWA International Academic Partnership Programme. This article/publication is based upon work from COST Action CA 17140 “Cancer Nanomedicine from the Bench to the Bedside” supported by COST (European Cooperation in Science and Technology) 2018-2022.

## Conflict of Interest

The authors declare that the research was conducted in the absence of any commercial or financial relationships that could be construed as a potential conflict of interest.

## Publisher’s Note

All claims expressed in this article are solely those of the authors and do not necessarily represent those of their affiliated organizations, or those of the publisher, the editors and the reviewers. Any product that may be evaluated in this article, or claim that may be made by its manufacturer, is not guaranteed or endorsed by the publisher.

## Supplementary Material

Supplementary Table 1 Differentially expressed miRNAs comparing HD and EARLY and LATE treated HIV-1 infected young adults.

## References

[1] UNAIDS. Global HIV & AIDS Statistics—2020 Fact Sheet. Geneva, Switzerland: UNAIDS (2020).

[2] Barlow-Mosha L Musiime V Davies M-A Prendergast AJ Musoke P Siberry G. Universal Antiretroviral Therapy for HIV-Infected Children: A Review of the Benefits and Risks to Consider During Implementation. J Int AIDS Society (2017) 20(1):21552. doi: 10.7448/IAS.20.1.21552 PMC5527851 28691434

[3] Domínguez-Rodríguez S Tagarro A Palma P Foster C Puthanakit T Jupimai T. Reduced Time to Suppression Among Neonates With HIV Initiating Antiretroviral Therapy Within 7 Days After Birth. JAIDS J Acquired Immune Deficiency Syndromes (2019) 82(5):483–90. doi: 10.1097/QAI.0000000000002188 PMC6857716 31714427

[4] Tagarro A Chan M Zangari P Ferns B Foster C De Rossi A. Early and Highly Suppressive Antiretroviral Therapy Are Main Factors Associated With Low Viral Reservoir in European Perinatally HIV-Infected Children. JAIDS J Acquired Immune Deficiency Syndromes (2018) 79(2):269–76. doi: 10.1097/QAI.0000000000001789 PMC6173292 30211778

[5] Rinaldi S Pallikkuth S Cameron M de Armas LR Cotugno N Dinh V. Impact of Early Antiretroviral Therapy Initiation on HIV-Specific CD4 and CD8 T Cell Function in Perinatally Infected Children. JI (2020) 204(3):540–9. doi: 10.4049/jimmunol.1900856 PMC6981070 31889024

[6] Ananworanich J Puthanakit T Suntarattiwong P Chokephaibulkit K Kerr SJ Fromentin R. Reduced Markers of HIV Persistence and Restricted HIV-Specific Immune Responses After Early Antiretroviral Therapy in Children. AIDS (2014) 28(7):1015–20. doi: 10.1097/QAD.0000000000000178 24384692

[7] Carrasco I Tarancon-Diez L Vázquez-Alejo E Jiménez de Ory S Sainz T Apilanez M. Innate and Adaptive Abnormalities in Youth With Vertically Acquired HIV Through a Multicentre Cohort in Spain. J Int AIDS Soc (2021) 24(10):e25804. doi: 10.1002/jia2.25804 34672108 PMC8528666

[8] Tarancón-Diez L Rull A Herrero P Vazquez-Alejo E Peraire J Guillén S. Early Antiretroviral Therapy Initiation Effect on Metabolic Profile in Vertically HIV-1-Infected Children. J Antimicrob Chemother (2021) 76(11):2993–3001. doi: 10.1093/jac/dkab277 34463735

[9] Dalzini A Petrara MR Ballin G Zanchetta M Giaquinto C De Rossi A. Biological Aging and Immune Senescence in Children With Perinatally Acquired HIV. J Immunol Res (2020) 2020:1–15. doi: 10.1155/2020/8041616 PMC7246406 32509884

[10] Colpaert RMW Calore M. Epigenetics and microRNAs in Cardiovascular Diseases. Genomics (2021) 113(2):540–51. doi: 10.1016/j.ygeno.2020.12.042 33482325

[11] Wang X He Y Mackowiak B Gao B. MicroRNAs as Regulators, Biomarkers and Therapeutic Targets in Liver Diseases. Gut (2021) 70(4):784–95. doi: 10.1136/gutjnl-2020-322526 33127832

[12] Wang P Qu X Zhou X Shen Y Ji H Fu Z. Two Cellular microRNAs, miR-196b and miR-1290, Contribute to HIV-1 Latency. Virology (2015) 486:228–38. doi: 10.1016/j.virol.2015.09.016 26469550

[13] Reynoso R Laufer N Hackl M Skalicky S Monteforte R Turk G. MicroRNAs Differentially Present in the Plasma of HIV Elite Controllers Reduce HIV Infection In Vitro. Sci Rep (2015) 4(1):5915. doi: 10.1038/srep05915 PMC4118195 25081906

[14] Heinson AI Woo J Mukim A White CH Moesker B Bosque A. Micro RNA Targets in HIV Latency: Insights Into Novel Layers of Latency Control. AIDS Res Hum Retroviruses (2021) 37(2):109–21. doi: 10.1089/aid.2020.0150 PMC7876363 33045840

[15] García-Merino I de las Cuevas N Jiménez JL García A Gallego J Gómez C. Pediatric HIV BioBank: A New Role of the Spanish HIV BioBank in Pediatric HIV Research. AIDS Res Hum Retroviruses (2010) 26(2):241–4. doi: 10.1089/aid.2009.0122 20156108

[16] Aguilera-Alonso D Sainz T Jimenez de Ory S Bernardino I Díez C Torres B. Clinical, Immunological, and Virological Outcomes Among Youths With Perinatal HIV After Transition to Adult Units in Spain From 1997 to 2016. JAIDS J Acquired Immune Deficiency Syndromes (2021) 86(2):240–7. doi: 10.1097/QAI.0000000000002539 33074855

[17] Liu Y Niu Y Li L Timani KA He VL Sanburns C. Tat Expression Led to Increased Histone 3 Tri-Methylation at Lysine 27 and Contributed to HIV Latency in Astrocytes Through Regulation of MeCP2 and Ezh2 Expression. J Neurovirol (2019) 25(4):508–19. doi: 10.1007/s13365-019-00751-0 PMC6750972 31020497

[18] Manganaro L Lusic M Gutierrez MI Cereseto A Del Sal G Giacca M. Concerted Action of Cellular JNK and Pin1 Restricts HIV-1 Genome Integration to Activated CD4+ T Lymphocytes. Nat Med (2010) 16(3):329–33. doi: 10.1038/nm.2102 20173753

[19] Zhang S-M Song M Yang T-Y Fan R Liu X-D Zhou P-K. HIV-1 Tat Impairs Cell Cycle Control by Targeting the Tip60, Plk1 and Cyclin B1 Ternary Complex. Cell Cycle (2012) 11(6):1217–34. doi: 10.4161/cc.11.6.19664 22391203

[20] Ryo A Tsurutani N Ohba K Kimura R Komano J Nishi M. SOCS1 is an Inducible Host Factor During HIV-1 Infection and Regulates the Intracellular Trafficking and Stability of HIV-1 Gag. Proc Natl Acad Sci (2008) 105(1):294–9. doi: 10.1073/pnas.0704831105 PMC2224204 18172216

[21] Deshmane SL Amini S Sen S Khalili K Sawaya BE. Regulation of the HIV-1 Promoter by HIF-1α and Vpr Proteins. Virol J (2011) 8(1):477 doi:10.1186/1743-422X-8-477 22023789 PMC3210103

[22] Uhl J Gujarathi S Waheed AA Gordon A Freed EO Gousset K. Myosin-X is Essential to the Intercellular Spread of HIV-1 Nef Through Tunneling Nanotubes. J Cell Commun Signal (2019) 13(2):209–24. doi: 10.1007/s12079-018-0493-z PMC6498342 30443895

[23] Li H Chi X Li R Ouyang J Chen Y. HIV-1-Infected Cell-Derived Exosomes Promote the Growth and Progression of Cervical Cancer. Int J Biol Sci (2019) 15(11):2438–47. doi: 10.7150/ijbs.38146 PMC6775309 31595161

[24] Kula A Guerra J Knezevich A Kleva D Myers MP Marcello A. Characterization of the HIV-1 RNA Associated Proteome Identifies Matrin 3 as a Nuclear Cofactor of Rev Function. Retrovirology (2011) 8(1):60. doi: 10.1186/1742-4690-8-60 21771346 PMC3160904

[25] Wang L Zhang H Solski PA Hart MJ Der CJ Su L. Modulation of HIV-1 Replication by a Novel RhoA Effector Activity. J Immunol (2000) 164(10):5369–74. doi: 10.4049/jimmunol.164.10.5369 PMC4435950 10799900

[26] Dong H Ye X Zhong L Xu J Qiu J Wang J. Role of FOXO3 Activated by HIV-1 Tat in HIV-Associated Neurocognitive Disorder Neuronal Apoptosis. Front Neurosci (2019) 13:44. doi: 10.3389/fnins.2019.00044 30778283 PMC6369160

[27] Fadel HJ Morrison JH Saenz DT Fuchs JR Kvaratskhelia M Ekker SC. TALEN Knockout of the PSIP1 Gene in Human Cells: Analyses of HIV-1 Replication and Allosteric Integrase Inhibitor Mechanism. J Virol (2014) 88(17):9704–17. doi: 10.1128/JVI.01397-14 PMC4136317 24942577

[28] Houzet L Yeung ML de Lame V Desai D Smith SM Jeang K-T. MicroRNA Profile Changes in Human Immunodeficiency Virus Type 1 (HIV-1) Seropositive Individuals. Retrovirology (2008) 5(1):118. doi: 10.1186/1742-4690-5-118 19114009 PMC2644721

[29] Seddiki N Brezar V Ruffin N Lévy Y Swaminathan S. Role of miR-155 in the Regulation of Lymphocyte Immune Function and Disease. Immunology (2013) 142(1):32–8 doi: 10.1111/imm.12227 PMC3992045 24303979

[30] Thai T-H Calado DP Casola S Ansel KM Xiao C Xue Y. Regulation of the Germinal Center Response by MicroRNA-155. Science (2007) 316(5824):604–8. doi: 10.1126/science.1141229 17463289

[31] Bignami F Pilotti E Bertoncelli L Ronzi P Gulli M Marmiroli N. Stable Changes in CD4+ T Lymphocyte miRNA Expression After Exposure to HIV-1. Blood (2012) 119(26):6259–67. doi: 10.1182/blood-2011-09-379503 22286198

[32] Violari A Cotton MF Gibb DM Babiker AG Steyn J Madhi SA. Early Antiretroviral Therapy and Mortality Among HIV-Infected Infants. N Engl J Med (2008) 359(21):2233–44. doi: 10.1056/NEJMoa0800971 PMC2950021 19020325

[33] Foster C Domínguez-Rodríguez S Tagarro A Gkouleli T Heaney J Watters S. The CARMA Study: Early Infant Antiretroviral Therapy—Timing Impacts on Total HIV-1 DNA Quantitation 12 Years Later. J Pediatr Infect Dis Soc (2021) 10(3):295–301. doi: 10.1093/jpids/piaa071 PMC8023306 32678875

[34] Jin C Cheng L Höxtermann S Xie T Lu X Wu H. MicroRNA-155 is a Biomarker of T-Cell Activation and Immune Dysfunction in HIV-1-Infected Patients. HIV Med (2017) 18(5):354–62. doi: 10.1111/hiv.12470 27981723

[35] Ayala-Suárez R Díez-Fuertes F Calonge E de la Torre Tarazona HE Gracia-Ruíz de Alda M Capa L. Insight in Mirnome of Long-Term Non-Progressors and Elite Controllers Exposes Potential RNAi Role in Restraining HIV-1 Infection. JCM (2020) 9(8):2452 doi: 10.3390/jcm9082452 PMC7464121 32751854

[36] Duette G Pereyra Gerber P Rubione J Perez PS Landay AL Crowe SM. Induction of HIF-1α by HIV-1 Infection in Infection in CD4+ T Cells Promotes Viral Replication and Drives Extracellular Vesicle-Mediated Inflammation. Sarkar SN, Smithgall TE. mBio (2018) 9(5):e00757-18 doi: 10.1128/mBio.00757-18 30206166 PMC6134101

[37] Martínez-Bonet M Puertas MC Fortuny C Ouchi D Mellado MJ Rojo P. Establishment and Replenishment of the Viral Reservoir in Perinatally HIV-1-Infected Children Initiating Very Early Antiretroviral Therapy. Clin Infect Dis (2015) 61(7):1169–78. doi: 10.1093/cid/civ456 PMC4560905 26063721

[38] Zhang L Chen J Cheng T Yang H Pan C Li H. Identification of Differentially Expressed Genes and miRNAs Associated With Esophageal Squamous Cell Carcinoma by Integrated Analysis of Microarray Data. BioMed Res Int (2020) 2020:1–16. doi: 10.1155/2020/2814548 PMC7352135 32714975

[39] Milush JM López-Vergès S York VA Deeks SG Martin JN Hecht FM. CD56negCD16+NK Cells are Activated Mature NK Cells With Impaired Effector Function During HIV-1 Infection. Retrovirology (2013) 10(1):158. doi: 10.1186/1742-4690-10-158 24351015 PMC3892122

[40] Chang Y Cui M Fu X Zhang L Li X Li L. MiRNA-155 Regulates Lymphangiogenesis in Natural Killer/T-Cell Lymphoma by Targeting BRG1. Cancer Biol Ther (2019) 20(1):31–41. doi: 10.1080/15384047.2018.1504721 30299211 PMC6343692

[41] Wei MF Gu ZS Zheng LL Zhao MX Wang XJ. Long non-Coding RNA GAS5 Promotes Natural Killer Cell Cytotoxicity Against Gastric Cancer by Regulating miR-18a. neo (2020) 67(05):1085–93. doi: 10.4149/neo_2020_191014N1034 32538667

[42] Zhang J Han X Hu X Jin F Gao Z Yin L. IDO1 Impairs NK Cell Cytotoxicity by Decreasing NKG2D/NKG2DLs *via* Promoting miR-18a. Mol Immunol (2018) 103:144–55. doi: 10.1016/j.molimm.2018.09.011 30268986

[43] Zawislak CL Beaulieu AM Loeb GB Karo J Canner D Bezman NA. Stage-Specific Regulation of Natural Killer Cell Homeostasis and Response Against Viral Infection by microRNA-155. Proc Natl Acad Sci (2013) 110(17):6967–72. doi: 10.1073/pnas.1304410110 PMC3637707 23572582

[44] Kared H Martelli S Tan SW Simoni Y Chong ML Yap SH. Adaptive NKG2C+CD57+ Natural Killer Cell and Tim-3 Expression During Viral Infections. Front Immunol (2018) 9:686. doi: 10.3389/fimmu.2018.00686 29731749 PMC5919961

[45] Rayner KJ Suárez Y Dávalos A Parathath S Fitzgerald ML Tamehiro N. MiR-33 Contributes to the Regulation of Cholesterol Homeostasis. Science (2010) 328(5985):1570–3. doi: 10.1126/science.1189862 PMC3114628 20466885

[46] Pedretti S Brulhart-Meynet M-C Montecucco F Lecour S James RW Frias MA. HDL Protects Against Myocardial Ischemia Reperfusion Injury *via* miR-34b and miR-337 Expression Which Requires STAT3. Gallyas F. PloS One (2019) 14(6):e0218432. doi: 10.1371/journal.pone.0218432 31220137 PMC6586303

[47] Bailey WJ Barnum JE Erdos Z LaFranco-Scheuch L Lane P Vlasakova K. A Performance Evaluation of Liver and Skeletal Muscle-Specific miRNAs in Rat Plasma to Detect Drug-Induced Injury. Toxicol Sci (2019) 168(1):110–25. doi: 10.1093/toxsci/kfy282 30496518

[48] Cárdenas-Bedoya J Marquez-Pedroza J Morán-Moguel MC Escoto-Delgadillo M Vázquez-Valls E González-Enríquez GV. MicroRNA-296-5p is Differentially Expressed in Individuals With and Without HIV-1 Infection. Genet Mol Biol (2020) 43(3):e20200017 doi: 10.1590/1678-4685-GMB-2020-0017 32584920 PMC7315763

[49] Price NL Singh AK Rotllan N Goedeke L Wing A Canfrán-Duque A. Genetic Ablation of miR-33 Increases Food Intake, Enhances Adipose Tissue Expansion, and Promotes Obesity and Insulin Resistance. Cell Rep (2018) 22(8):2133–45. doi: 10.1016/j.celrep.2018.01.074 PMC5860817 29466739

