# Supplementary material for: miRNA Profile Based on ART Delay in Vertically Infected HIV-1 Youths Is Associated With Inflammatory Biomarkers and Activation and Maturation Immune Levels
Source: Front Immunol. 2022 Apr 22;13:878630. doi: 10.3389/fimmu.2022.878630 (PMC9074828; doi:10.3389/fimmu.2022.878630)
Supplement: Supplementary Table 1 — Differentially expressed miRNAs comparing HD and EARLY and LATE treated HIV-1 infected young adults. [file Table_1.docx]

Supplementary Material

# Supplementary Data

**Supplementary table 1. Differentially expressed miRNAs comparing HD and EARLY and LATE treated HIV-1 infected young adults.**

|  | **miRNA** | **Relative Quantity Ratio (HIV-1 young adults vs HD)** |  | **miRNA** | **Relative Quantity Ratio (HIV-1 young adults vs HD)** |
| --- | --- | --- | --- | --- | --- |
| **EARLY treated HIV-1 young adults VS HD** | hsa-miR-19a-3p | 4.031 | **LATE treated HIV-1 young adults VS HD** | hsa-miR-19a-3p | 2.913 |
|  | hsa-miR-484 | 0.497 |  | hsa-miR-484 | 0.506 |
|  | hsa-miR-23a-3p | 2.782 |  | hsa-miR-23a-3p | 2.309 |
|  | hsa-miR-328-3p | 0.399 |  | hsa-miR-29c-5p | 0.523 |
|  | hsa-miR-16-5p | 8.674 |  | hsa-miR-302b-3p | 0.154 |
|  | hsa-miR-21-5p | 6.057 |  | hsa-miR-328-3p | 0.453 |
|  | hsa-miR-24-3p | 4.745 |  | hsa-miR-92a-3p | 2.974 |
|  | hsa-miR-302b-3p | 0.093 |  | hsa-miR-181c-3p | 0.348 |
|  | hsa-miR-361-3p | 2.517 |  | hsa-miR-16-5p | 6.82 |
|  | hsa-miR-92a-3p | 3.38 |  | hsa-miR-24-3p | 3.71 |
|  | hsa-miR-501-3p | 0.093 |  | hsa-miR-21-5p | 4.1 |
|  | hsa-miR-941 | 3.308 |  | hsa-miR-132-3p | 0.489 |
|  | hsa-miR-19b-3p | 2.291 |  | hsa-miR-2110 | 0.252 |
|  | hsa-miR-25-3p | 1.819 |  | hsa-miR-19b-3p | 1.82 |
|  | hsa-miR-361-5p | 5.178 |  | hsa-miR-941 | 2.2 |
|  | hsa-miR-132-3p | 0.506 |  | hsa-miR-424-3p | 4.479 |
|  | hsa-miR-222-3p | 3.044 |  | hsa-miR-25-3p | 1.618 |
|  | hsa-miR-27a-3p | 2.192 |  | hsa-miR-361-5p | 3.998 |
|  | hsa-miR-532-5p | 2.7 |  | hsa-miR-133a-3p | 0.411 |
|  | hsa-miR-150-5p | 3.434 |  | hsa-miR-448 | 0.211 |
|  | hsa-miR-2110 | 0.286 |  | hsa-miR-93-5p | 2.749 |
|  | hsa-miR-424-3p | 4.245 |  | hsa-miR-222-3p | 2.259 |
|  | hsa-miR-664a-3p | 2.208 |  | hsa-miR-181a-5p | 0.707 |
|  | hsa-miR-155-5p | 3.822 |  | hsa-miR-501-3p | 0.27 |
|  | hsa-miR-500a-5p | 3.673 |  | hsa-miR-17-5p | 2.95 |
|  | hsa-miR-93-3p | 0.529 |  | hsa-miR-223-3p | 2.216 |
|  | hsa-miR-17-5p | 3.519 |  | hsa-miR-664a-3p | 1.679 |
|  | hsa-miR-223-3p | 2.551 |  | hsa-miR-27a-3p | 1.737 |
|  | hsa-miR-93-5p | 3.012 |  |  | |
|  | hsa-miR-133a-3p | 0.429 |  |  |  |
|  | hsa-miR-26a-5p | 3.436 |  |  |  |
|  | hsa-miR-29b-3p | 2.586 |  |  |  |
|  | hsa-miR-494-3p | 0.161 |  |  |  |
|  | hsa-miR-22-5p | 1.758 |  |  |  |
